# A group I metabotropic glutamate receptor controls synaptic gain between rods and rod bipolar cells in the mouse retina

**DOI:** 10.14814/phy2.13885

**Published:** 2018-10-18

**Authors:** Chase B. Hellmer, Melissa Rampino Clemons, Scott Nawy, Tomomi Ichinose

**Affiliations:** ^1^ Department of Ophthalmology, Visual and Anatomical Sciences Wayne State University School of Medicine Detroit Michigan 48201; ^2^ Dominic P Purpura Dept. of Neuroscience Albert Einstein College of Medicine Bronx Bronx New York 10461; ^3^ Department of Ophthalmology and Visual Sciences University of Nebraska Medical Center Omaha Nebraska 68198

**Keywords:** Light response, metabotropic glutamate receptors, retina

## Abstract

The canonical mGluR6‐Trpm1 pathway that generates the sign‐inverting signal between photoreceptors and ON bipolar cells has been well described. However, one type of ON bipolar cell, the rod bipolar cell (RBC), additionally is thought to express the group I mGluRs whose function is unknown. We examined the role of group I mGluRs in mouse RBCs and here provide evidence that it controls synaptic gain between rods and RBCs. In dark‐adapted conditions, the mGluR1 antagonists LY367385 and (RS)‐1‐Aminoindan‐1,5‐dicarboxylic acid, but not the mGluR5 antagonist 2‐Methyl‐6‐(phenylethynyl)pyridine hydrochloride reduced the light‐evoked responses in RBCs indicating that mGluR1, but not mGluR5, serves to potentiate RBC responses. Perturbing the downstream phospholipase C (PLC)‐protein kinase C (PKC) pathway by inhibiting PLC, tightly buffering intracellular Ca^2+^, or preventing its release from intracellular stores reduced the synaptic potentiation by mGluR1. The effect of mGluR1 activation was dependent upon adaptation state, strongly increasing the synaptic gain in dark‐, but not in light‐adapted retinas, or in the presence of a moderate background light, consistent with the idea that mGluR1 activation requires light‐dependent glutamate release from rods. Moreover, immunostaining revealed that protein kinase C*α* (PKC *α*) is more strongly expressed in RBC dendrites in dark‐adapted conditions, revealing an additional mechanism behind the loss of mGluR1 potentiation. In light‐adapted conditions, exogenous activation of mGluR1 with the agonist 3,5‐Dihydroxyphenylglycine increased the mGluR6 currents in some RBCs and decreased it in others, suggesting an additional action of mGluR1 that is unmasked in the light‐adapted state. Elevating intracellular free Ca^2+^, consistently resulted in a decrease in synaptic gain. Our results provide evidence that mGluR1 controls the synaptic gain in RBCs.

## Introduction

The retina encodes and transmits visual information over a range of light intensities spanning approximately 12 log units. To accommodate this range, the retina is organized into multiple circuits that are selectively responsive to scotopic (dim light) and photopic (bright light) stimuli. The primary rod pathway is the predominant circuit for transmission of visual information in low light intensities (Kolb and Famiglietti 1974; Volgyi et al. 2004). In this pathway, light signals detected by rods are converted to electrical signals that are transmitted to rod bipolar cells (RBCs), then to AII amacrine cells, ON cone bipolar cells, and ganglion cells. Though initially thought to be independent (Aguilar and Stiles 1954), the dim‐light driven rod pathway and the bright‐light driven cone pathway are now believed to jointly participate in the mesopic, or mid, range of vision (Verweij et al. 1999; Xin and Bloomfield 1999; Volgyi et al. 2004). This means that the intensity and wavelength of the light determine the degree to which the rod and cone pathways contribute to transmitting visual signals to downstream ganglion cells.

Because visual activity occurs over such a wide range of light levels, transmission in the retina requires gain control mechanisms that maximize the ability of the system to reliably relay signals at all light intensities. There is evidence for mechanisms of gain control at the level of the photoreceptors (Schneeweis and Schnapf 2000; Dunn and Rieke 2006), at the synapse between RBCs and AII amacrine cells (Dunn and Rieke 2006, 2008; Dunn et al. 2006; Jarsky et al. 2011; Oesch and Diamond 2011), and further downstream at the ganglion cells (Schwartz and Rieke 2013). These mechanisms control gain both by amplifying low intensity signals and by decreasing noise that is inherent in the system. A primary gain control mechanism should exist at the first visual synapse, between rods and RBCs; however, it has not been identified because of the complicated transmission system of the mGluR6‐TrpM1 complex. Neurons that receive direct synaptic input from photoreceptors must have the ability to filter out noise and amplify small signals to ensure that accurate and relevant information is conveyed downstream.

Here, we define a novel gain control mechanism at the rod‐RBC synapse that regulates signal transmission. When the number of available photons is low, it amplifies light signals, whereas in mesopic conditions, it can suppress rod inputs during the signal transition to cone dominancy. This mechanism requires activation of the group I metabotropic glutamate receptor, mGluR1, which has been found at the dendritic tips of RBCs in the rat retina (Koulen et al. 1997). In scotopic conditions, continuous glutamate release from rods activated the mGluR1 to amplify mGluR6‐TrpM1 mediated synaptic inputs through the phospholipase C (PLC)‐phosphatidylinositol 4,5‐bisphosphate (PIP_2_)‐protein kinase C (PKC) signaling pathway. When background light levels were increased, mGluR1 exhibited diverse effects, including suppressing synaptic inputs. This form of plasticity may function as a mechanism to regulate signaling in the primary rod pathway.

## Materials and Methods

### Ethical approval

All animal protocols were approved by the Institutional Animal Care and Use Committees at Wayne State University (protocol no. A05‐03‐15) and Albert Einstein College of Medicine (protocol no. 20130807). All necessary steps were taken to minimize animal suffering. The tissues were harvested immediately after the animal was killed. Euthanasia was conducted using a combination of carbon dioxide and cervical dislocation, or isoflurane and cervical dislocation.

### Retinal preparation

Patch clamp experiments on rod bipolar cells were carried out in parallel by the Ichinose and Nawy laboratories. The experimental techniques were similar to those described previously (Rampino and Nawy 2011; Ichinose and Lukasiewicz 2012; Ichinose et al. 2014) and are summarized below. Mice (28–60 day of age; 10–30 g, male and female, C57BL/6J strain from The Jackson Laboratory, or C57BL/6 from Charles River Laboratories) were dark‐adapted overnight and were euthanized in the dark using an infrared apparatus. Using a dissecting microscope with infrared viewers, the cornea and the lens were quickly removed and the retina was isolated. The retina slab was placed on a piece of filter membrane (HABG01300, Millipore) and cut into slice preparations (150–250 *μ*m thick) using a hand‐made chopper or a manual tissue slicer (Stoelting Co). Only the dorsal half of the retina was used. The dissection medium was cooled and continuously oxygenated. The retinal preparations were stored in an oxygenated dark box at room temperature.

### Patch clamp recordings

Whole cell patch recordings were made from bipolar cell somas in retinal slices by viewing them with an upright microscope (Slicescope Pro 2000, Scientifica) equipped with a CCD camera (Retiga‐2000R, Q‐Imaging) or with an Olympus BM51 with a 40X water immersion objective using a monochrome CCD camera (Cohu 4912). Patch pipettes were pulled from fire polished capillary tubing (World Precision Instruments) to resistance of 5–10 MΩ using either a P1000 Puller (Sutter Instruments) or a two‐stage vertical puller (Narishige, Model # PP‐830). Recordings were obtained with a Multi Clamp 700B or an Axopatch 200B amplifier (Molecular Devices), and digitized with an Axon Digidata 1440A or an ITC‐18 analog‐to‐digital converter. Data were filtered at 1–2 kHz and digitized at 2–5 kHz. Recordings had input resistances of approximately 1 GΩ and series resistance of 5–20 MΩ, which was not compensated for. Perforated patch recordings were made by the inclusion of 0.5 mg/mL Amphotericin B (Sigma, cat no. A9528), which were verified by sulforhodamine fluorescent dye in the recording pipettes that did not label the soma of the recorded cells. The voltage clamp recordings were conducted at the equilibrium potential for chloride of −40 mV, or +38 mV. Current clamp recordings were carried out using perforated patch clamping at the resting membrane potential. All recordings were performed at 30–34°C. Liquid junction potentials were corrected after each recording. Rod bipolar cells were morphologically identified by the inclusion of 0.005% sulforhodamine B (Sigma) or AlexaFluor 488 (50 *μ*mol/L) in pipette solution. For clarity, the voltage clamp recordings have been inverted in all figures so as to appear inward.

### Solutions and drugs

**Figure 1:**
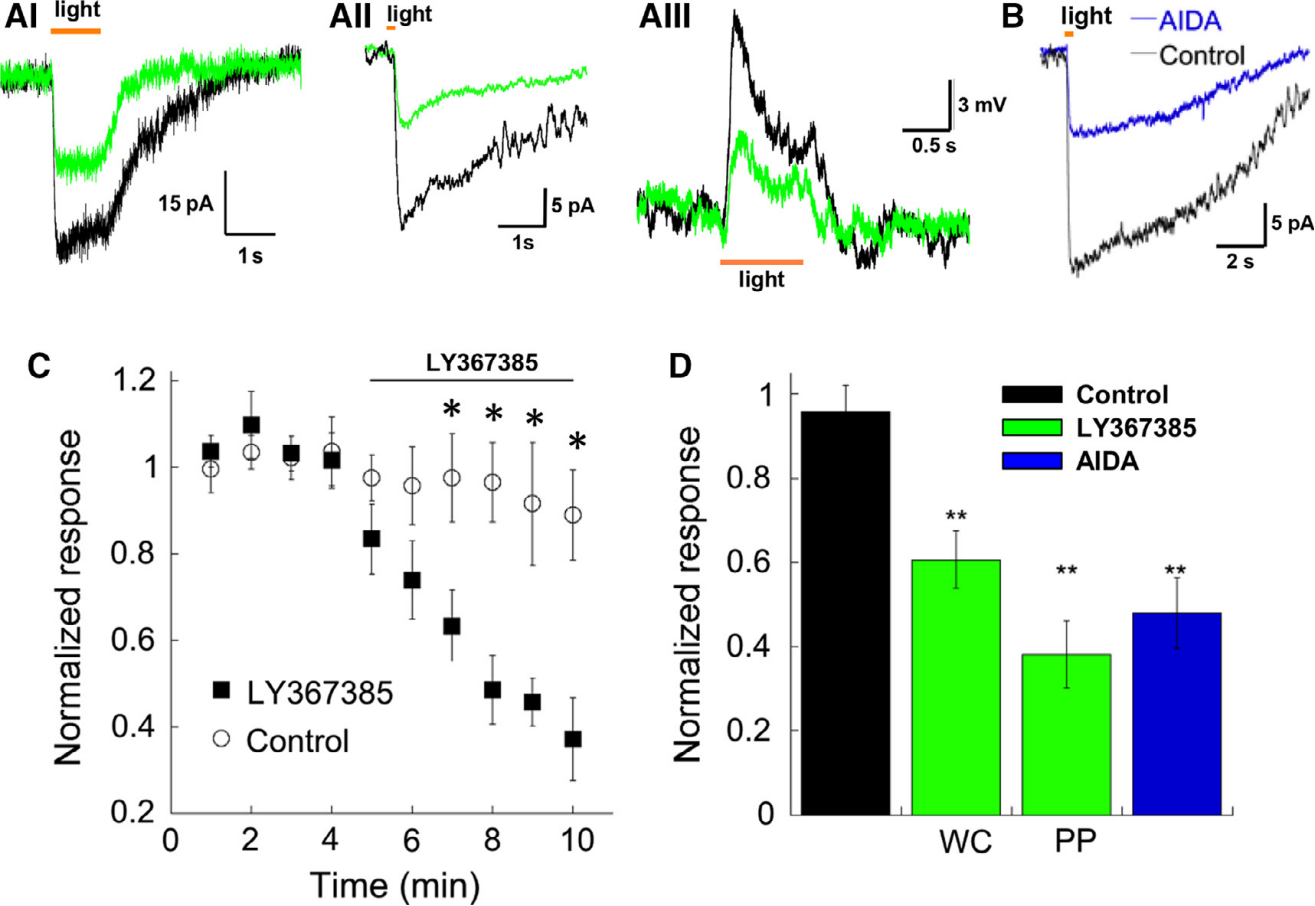
Light‐evoked excitatory postsynaptic currents and potentials (L‐EPSCs and L‐EPSPs) are reduced by mGluR1 antagonists. (A) Representative traces showing the effect of LY367385 on the light response of the rod bipolar cell (RBCs) voltage clamped at −40 mV (i), +40 mV (ii) and in current clamp using the perforated patch configuration (iii). (B) Example of the inhibition by (RS)‐1‐Aminoindan‐1,5‐dicarboxylic acid (AIDA) on the L‐EPSC of another RBC held at +40 mV. Currents are all shown as inward for consistency between panels. (C) The time course of LY367385 inhibition of L‐EPSPs. Data are for perforated patch experiments (*n* = 6, *P < 0.05). Control experiments in which no drug was applied are shown for comparison (*n* = 3 for perforated patch, *n* = 4 for whole cell recording). (D) A summary graph showing the effects of LY367385 and AIDA on light responses under different conditions after 5 min of drug application. Control (no drug application): *n* = 7, LY367385, whole cell (WC) *n* = 9, *P* = 0.002; perforated patch (PP), *n* = 6 and *P* = 0.0002; AIDA (+40 mV), *n* = 6, *P* = 0.001.

**Figure 2:**
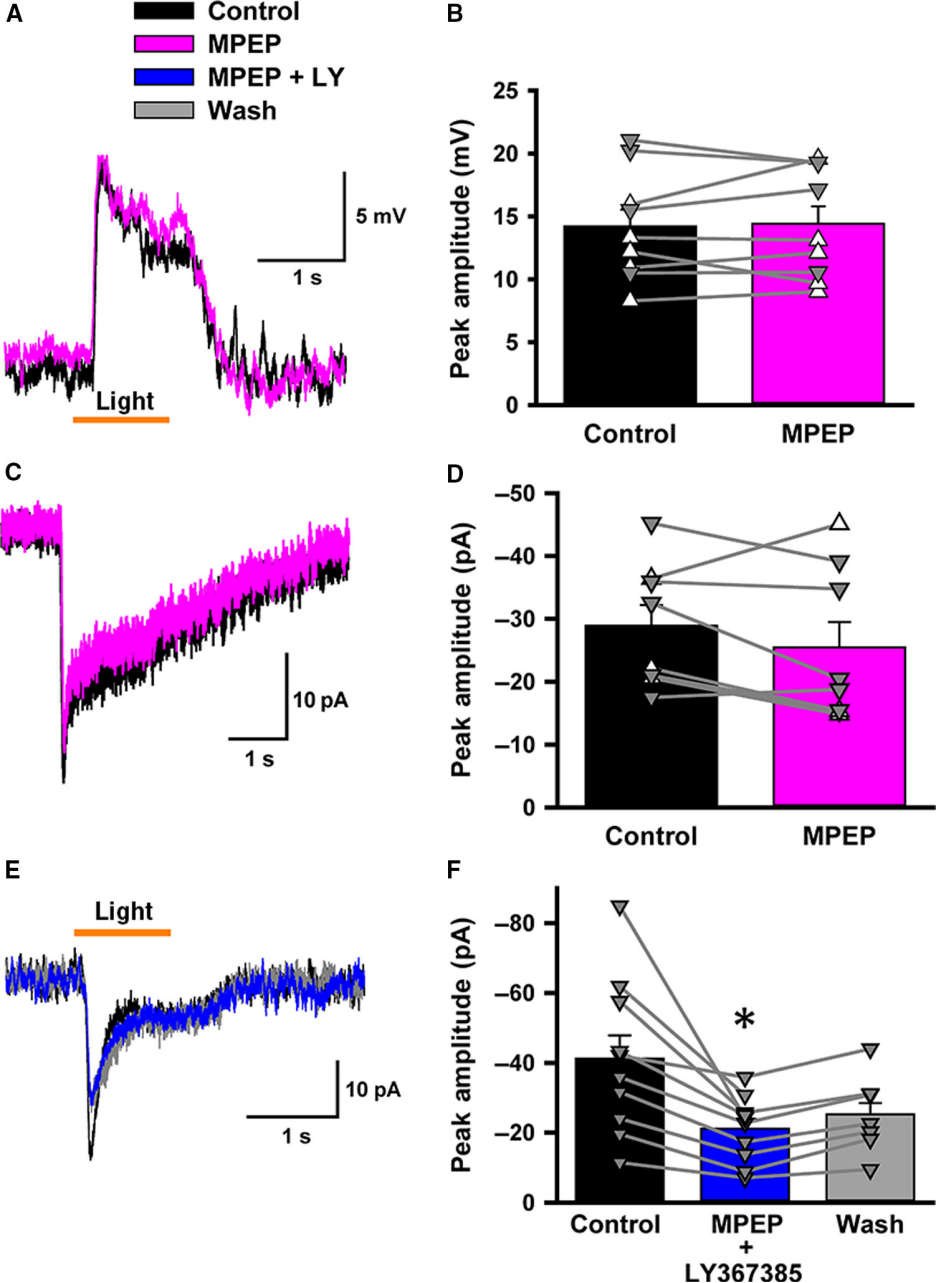
2‐Methyl‐6‐(phenylethynyl)pyridine hydrochloride (MPEP) did not affect light responses in rod bipolar cells (RBCs). (A) A light‐evoked excitatory postsynaptic potential (L‐EPSP) (black) was unaffected by application of an mGluR5 antagonist, MPEP (50 *μ*mol/L, pink). (B) A summary graph showing the effects of MPEP on RBC L‐EPSPs (*n* = 9, *P* > 0.1). In this and following panels, open triangles (∆) indicate perforated patch and closed triangles (▼) indicate whole cell recordings. (C) Similarly, MPEP did not affect RBC L‐EPSCs. (D) A summary graph showing the effects of MPEP on RBC L‐EPSCs (*n* = 8, *P* > 0.1). (E) A dim light‐evoked excitatory postsynaptic current (EPSC) (black) was reduced by co‐application of MPEP (50 *μ*mol/L) and LY367385 (100 *μ*mol/L) (blue). (F) A summary graph showing the effects of MPEP and LY367385 co‐application on L‐EPSCs (*n* = 10, paired *t*‐test, *P* < 0.01).

Retinal dissections were performed in HEPES‐buffered extracellular solution containing the following (in mmol/L): 115 NaCl, 2.5 KCl, 2.5 CaCl_2_, 1.0 MgCl_2_, 10 HEPES, 28 glucose, adjusted to pH 7.38 by NaOH or in Ames’ media (Sigma) continuously bubbled with 95% O_2_ and 5% CO_2_. Physiological recordings were performed in Ames’ medium buffered with NaHCO_3_, which was bubbled with 95% O_2_ and 5% CO_2_; the pH was 7.4 at 30°C. The intracellular solution contained the following (in mM): 100 Cs‐gluconate, 10 HEPES, 0.5 EGTA, 4 NaCl, 0.5 MgCl_2_, 15 mmol/L TEA‐Cl, 5 ATP‐Mg, and 0.5 GTP‐Na, adjusted to pH 7.2 with CsOH (Figs. 1, 2A, 5, 6, 7, 8) or 100 Cs‐gluconate, 40 HEPES, 2.5 EGTA, 4 Mg‐ATP, and 1 Li‐GTP, adjusted to pH 7.3 using cesium hydroxide. Cs‐gluconate was replaced with K‐gluconate and TEA‐Cl was removed when recording in current clamp mode (Figs. 1C and 2A). A cocktail of inhibitory receptor antagonists, including a glycine receptor antagonist, strychnine (1 *μ*mol/L, Sigma), a GABA_A_ receptor antagonist, (−)‐bicuculline methobromide (50 *μ*mol/L; Axxora), and a GABA_C_ receptor antagonist, TPMPA (Tocris Bioscience, cat. no. 1040) (50 *μ*mol/L) were bath applied continuously for all recordings to suppress the network effect. Dark conditions were imitated with bath perfusion of the mGluR6 agonist L‐AP4 (4 *μ*mol/L, Tocris Bioscience, cat. # 0103) while the light stimulation was mimicked with the mGluR6 antagonist (RS)‐α‐Cyclopropyl‐4‐phosphonophenylglycine (CPPG) (600 *μ*mol/L, Tocris Bioscience, cat. # 0972). Group I metabotropic receptors were stimulated with the group I agonist (S)‐3,5‐Dihydroxyphenylglycine (DHPG) (100 *μ*mol/L, Tocris Bioscience, cat. # 0805). Antagonists for mGluR1*α*, LY367385 (100 *μ*mol/L, Tocris Bioscience, cat. # 1237), and (RS)‐1‐Aminoindan‐1,5‐dicarboxylic acid (AIDA) (100 *μ*mol/L, Sigma, A254), and an mGluR5*α* antagonist, 2‐Methyl‐6‐(phenylethynyl)pyridine hydrochloride (MPEP) (50 *μ*mol/L, Tocris Bioscience, cat. # 1212) were included in the bath solution during light stimulation. DHPG, LY367385, and AIDA have *K*_d_ values of 1.4 *μ*mol/L, 1.1 *μ*mol/L, and 98 *μ*mol/L respectively (Lavreysen et al. 2003) while MPEP has an IC_50_ of 36 nmol/L (Gasparini et al. 1999). LY341495 (500 *μ*mol/L, cat. # 1209), U73122 (10 *μ*mol/L, cat. # 1268), xestospongin C (1 *μ*mol/L, cat. # 1280) were purchased from Tocris Bioscience, while 1‐Oleoyl‐2‐acetyl‐sn‐glycerol (OAG) (100 *μ*mol/L, cat. # O6754) was purchased from Sigma‐Aldrich.

### Light stimulation

Green light (500 nm) was generated by a 25 watt halogen bulb with an interference filter and a series of neutral density filters inserted into the light path. The light was projected through a 60X or 40X objective lens at photoreceptors in the vicinity of recording bipolar cells with a diameter of 100 *μ*m, which is slightly larger than the size of receptive field center of a bipolar cell (Berntson and Taylor 2000; Borghuis et al. 2013). An intensity of ~1.0 × 10^3^ photons · *μ*m^−2^ · sec^−1^ was used for brief light stimulation, while a background intensity of 1.4 × 10^3^ photons · *μ*m^−2^ · sec^−1^ was used for the continuous background in Figure 5.

### Immunohistochemistry

Retinal cryosections were used for PKC immunohistochemistry. For making semi‐thin sections, retinal whole‐mounts were fixed with 4% paraformaldehyde in 0.1 mol/L phosphate buffer (PB) solution, pH 7.4, for 60 min at room temperature. For dark‐adapted tissue, animals were kept in a dark box for 12 h prior to tissue harvesting. All procedures were done up to tissue fixation without exposing the samples to light. For light‐adapted tissue, mice were adapted at room light for 6 h and dissection was performed in light conditions. After fixation, retinas were rinsed several times in 0.1 mol/L PB, and cryoprotected in 30% sucrose overnight at 4°C. Prior to embedding, retinas were placed in a 3:1 mixture of 30% sucrose solution and Tissue Freezing Medium (EMS) for 1 h. The tissue was embedded in Tissue Freezing Medium and was sectioned at 14 *μ*m with a Micron HM 525 cryostat (Thermo Scientific). Cryosections were collected on SuperFrost Plus glass slides (No. 12‐550‐15, Fisher Scientific).

Retinal thick sections (~250 μm) were used for mGluR1 staining. Rod bipolar cells in these slice preparations were filled with Neurobiotin (0.5%, Vector Lab) using the patch clamp technique. We did not use the PKC antibody for RBC staining because both mGluR1 and PKC primary antibodies are raised in mouse. As the mGluR1 antibody was reported not to work under our normal conditions of 4% PFA fixation (Ayala et al. 2012), slices were instead fixed with 2% paraformaldehyde for 1 h. Antigen retrieval was then performed by applying a citrate buffer (10 mmol/L citric acid and 0.1% Tween‐20 in _dd_H_2_O, pH 6.0) at 80°C for 15 min, followed by washing with 0.1 mol/L PB solution three times for 15 min each.

Retina sections were blocked in a solution containing 10% normal donkey serum (NDS) and 0.5% Triton‐X in phosphate buffered saline (PBS) for 1 h. All following procedures were performed at room temperature. Primary antibodies were diluted in 3% NDS and 0.5% Triton‐X in PBS. A protein kinase Cα (PKC*α*) antibody (Novus Biologicals, 600‐201, RRID: AB_2284346) and a mGluR6 antibody (Neuromics, GP13105, RRID: AB_2341540) were used to stain thin sections while a mGluR1 antibody (1:100, BD Biosciences, Cat# 556389, RRID:AB_396404) and streptavidin‐conjugated Alexa 568 (1:500, Invitrogen, S11226) were used to stain thick sections. All antibodies were centrifuged at 12,000*g* for 5 min prior to use. PKC stained sections were incubated with primary antibodies for 2 h, followed by a mixture of secondary antibodies conjugated with Alexa dyes (Life Technologies, Inc.) for 1 h. The stained thick sections of mGluR1 were incubated with primary antibodies overnight, followed by a secondary antibody conjugated with an Alexa dye (Life Technologies, Inc.) for 2 h. The preparations were viewed with a confocal microscope (Leica, TCS SP8) using a 40x oil immersion objective lens or a 63x water immersion objective lens. Laser intensity, detector wavelength, and all other settings were identical across the samples. Images were captured by digital sectioning at 0.3 *μ*m thick. Sequential scanning was used to eliminate crosstalk between channels and to better separate signals from each other.

### Western Blot

Retinal tissue was lysed by sonication in radio‐immunoprecipitation assay (RIPA) buffer with a protease inhibitor cocktail (P8340, Sigma) including 0.5 *μ*mol/L 4‐(2‐aminoethyl) benzenesulfonyl fluoride hydrochloride (AEBSF) 0.4 *μ*mol/L aprotinin, 10 *μ*mol/L leupeptin, 20 *μ*mol/L bestatin, 7.5 *μ*mol/L pepstatin A, and 7.0 *μ*mol/L E‐64. Samples were sonicated followed by centrifugation at 12,000*g* for 20 min. Total protein was quantified by Micro BCA protein assay kit (23235, ThermoFisher). Total protein samples (30 *μ*g) were run on an 8% SDS‐PAGE in Tris‐glycine‐SDS buffer and then electro‐blotted onto a nitrocellulose membrane (BioRad). After blocking with 5% bovine serum albumin (BSA) in Tris‐buffered saline containing 0.05% Tween‐20 and 5% milk (TBS‐T) for 1 h, the membrane was probed with the mGluR1 primary antibody (1:1000) in 3% BSA in TBS‐T overnight at 4°C. After three washes with TBS‐T, the membrane was incubated with a HRP secondary antibody (1:2000) diluted in TBS‐T with 5% milk at room temperature for 2 h. Bands were developed with Supersignal West Femto Chemiluminescent Substrate (34095, ThermoFisher Scientific) and visualized using a Kodak image station, 4000R Pro Molecular Imaging System (Carestream Health Inc,. Rochester, NY).

### Statistics and data analysis

We measured the amplitude and time course of CPPG and light‐evoked responses using Clampfit or Axograph X and Kaleidagraph. We measured the peak amplitude (10–90%) from the baseline potential (before light onset). For pharmacological experiments, EPSC amplitudes of onset responses were analyzed at various time points before and after application of drugs. Values are presented as mean ± SEM. Statistical significance was determined using unpaired (two tailed) *t* tests, unless indicated otherwise. Differences were considered significant if *P* < 0.05. In some cases, significance was determined using a paired *t* test as indicated in the figure legends.

Confocal images were analyzed using Adobe Photoshop (Version 13.0, Adobe Systems, Inc.). Arbitrary fluorescent intensity for PKC labeling was measured at three different regions of interest (ROI): outer plexiform layer (OPL), inner nuclear layer (INL), and axon terminals, and compared across conditions (Figure 6). When slices were tilted, multiple images were selected for analyzing the different ROIs. Using the histogram function of Adobe Photoshop, the mean pixel intensity was analyzed for each ROI. Mean values were averaged across multiple animals to provide final values.

## Results

### mGluR1, but not mGluR5 antagonists reduce L‐EPSCs and L‐EPSPs in RBCs

There are two subtypes of group I mGluRs, mGluR1 and mGluR5, and both have been reported to be expressed on the dendrites of RBCs in the rat retina (Koulen et al. 1997), yet the function of these receptors has not been characterized. Because they are expressed in RBC dendrites, they may play a role in the mGluR6 cascade. Although these two receptors are assumed to initiate the same signaling cascade, their effects are not always identical (Viwatpinyo and Chongthammakun 2009; Kramer and Williams 2015). We therefore tested the effects of mGluR1 and mGluR5 antagonists on light‐evoked excitatory postsynaptic currents and potentials (L‐EPSCs and L‐EPSPs) in dark‐adapted conditions where glutamate was continuously released from photoreceptors.

The addition of the mGluR1 antagonist LY367385 (100 *μ*mol/L) to the bath solution reduced the L‐EPSC to 61 ± 6% of the initial response (Fig. 1Ai). As RBC responses may be a subject to rundown, particularly at negative holding potentials (Nawy 2000, 2004; Berntson et al. 2004), we then performed this experiment at positive holding potentials. LY367385 also strongly reduced the amplitude of the L‐EPSCs at +40 mV (Fig. 1Aii). As there was no significant difference between the amount of block at negative vs. positive holding potentials, the results have been pooled. Application of LY367385 also reduced the L‐EPSPs obtained in current clamp using perforated patch clamp (Fig. 1Aiii). Application of AIDA, another mGluR1 antagonist, also decreased the amplitude of the light response to 54 ± 8% of its initial value (Fig. 1B). As shown in the summary, time course for perforated patch recordings, the effect of LY367385 is not due to rundown as the voltage reductions started after the application of the drug, and were absent in control cells (Fig. 1C). The results are summarized in Figure 1D. Both antagonists, LY367385 and AIDA, consistently reduced response amplitudes, indicating that endogenous mGluR1 activation enhances synaptic currents in dark‐adapted conditions.

Next, we examined mGluR5, the other group I mGluR subtype. We tested the effect of the mGluR5 antagonist, MPEP (50 *μ*mol/L), on L‐EPSPs in the dark‐adapted state. MPEP did not change the L‐EPSP amplitudes either in whole cell or perforated patch recordings (Fig. 2A–B). Similarly, MPEP application did not change L‐EPSCs (Fig. 2C–D). In addition, the effect of co‐application of MPEP and LY367385 on light‐evoked current was indistinguishable from the effect of LY367385 alone (Fig. 2E–F). Finally, we observed that neither the application of MPEP or the LY367385 shifted the membrane potential of RBCs (data not shown).These results suggest that mGluR5 does not participate in regulation of rod–RBC synaptic transmission.

### PKC mediates the effects of group I mGluRs on RBC currents

**Figure 3:**
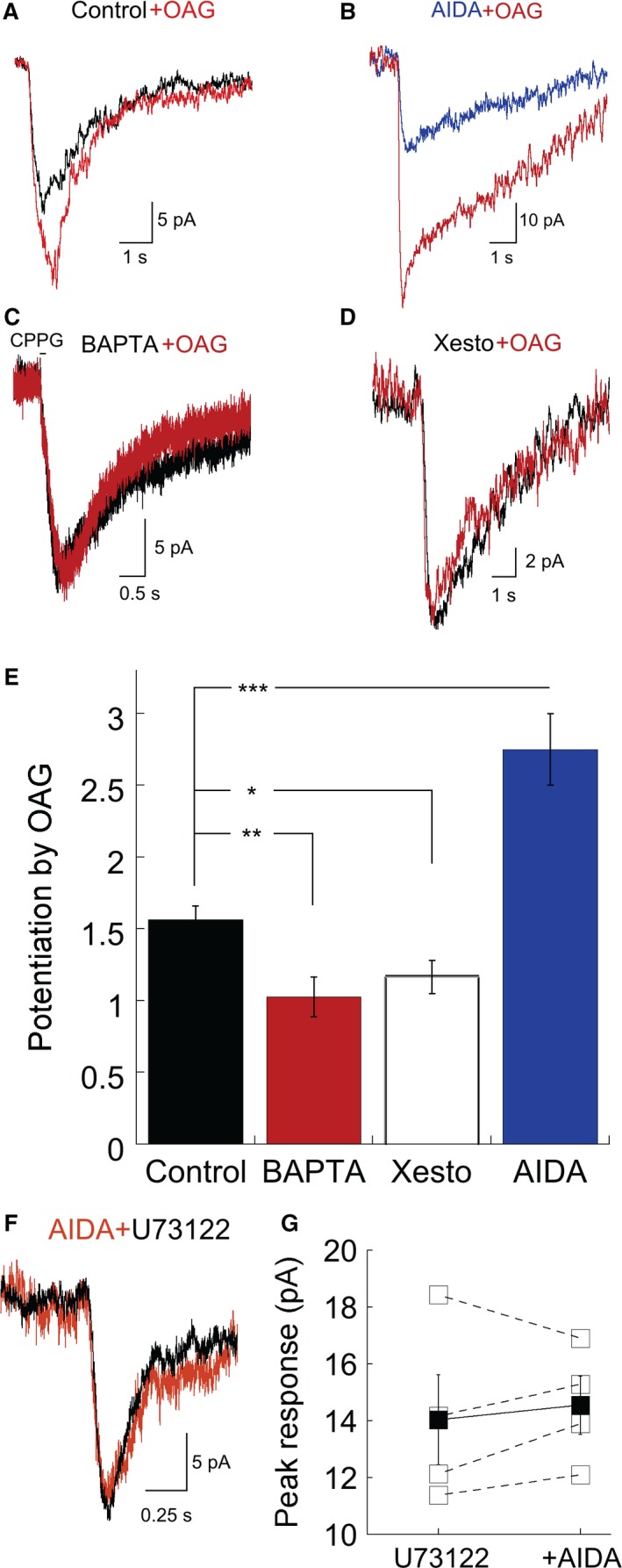
Phospholipase C (PLC)‐phosphatidylinositol 4,5‐bisphosphate(PIP_2_)‐protein kinase C (PKC) signaling mediates the effects of mGluR1 on mGluR6‐evoked currents. Raw traces depicting the effect of PKC stimulation on the rod bipolar cell (RBC) light response under four different conditions. (A) Light response of an RBC at break‐in and after dialysis with the PKC activator 1‐Oleoyl‐2‐acetyl‐sn‐glycerol (OAG). (B) As in (A) except that (RS)‐1‐Aminoindan‐1,5‐dicarboxylic acid (AIDA) was added to the bath prior to the experiment to block mGluR1 function. (C) Trpm1 current evoked at break‐in and after OAG dialysis in an internal solution containing 10 mmol/L BAPTA rather than EGTA. A pharmacological approach was used to activate Trpm1 current (see Methods). (D) Light‐evoked Trpm1 currents at break‐in (black trace) and after 5 min of dialysis with OAG and the IP_3_ receptor inhibitor xestospongin C. (E) Summary data of the four experiments depicted in A–D (*n* = 5 for all conditions. ***P = 0.006, **P = 0.01, *P = 0.03). (F) Sample traces of Trpm1 current evoked by light in a cell dialyzed with the PLC inhibitor U73122 (10 *μ*mol/L) before and after bath application of AIDA. (G) Comparison of the response amplitudes after dialysis with U73122 and subsequent bath application of AIDA. Open and closed squares represent individual cells and the mean response respectively. AIDA application caused no significant change in response amplitude (*n* = 4; *P* > 0.1, paired *t* test comparing U73122 alone vs. U73122+AIDA).

The group I mGluRs couple to the PLC‐PKC pathway via G*α*_q_/G*α*_11_ (Hirono et al. 2001; Valenti et al. 2002). In RBCs, activation of PKC*α* increases and speeds‐up mGluR6‐evoked currents (Strauss et al. 2010; Rampino and Nawy 2011; Xiong et al. 2015). As such, we examined whether mGluR1 increased mGluR6‐currents through PIP_2_‐PLC‐PKC signaling. In the dark‐adapted retina, OAG (100 *μ*mol/L), a DAG analogue and a PKC activator with Ca^2+^, slightly potentiated the mGluR6‐evoked currents (Fig. 3A). This potentiation was strongly increased when mGluR1 receptors were blocked with the addition of AIDA, an mGluR1 antagonist, to the bath prior to recording (Fig. 3B), consistent with the idea that in darkness glutamate binds to mGluR1, continuously driving the PIP_2_‐PLC‐PKC pathway. Driving the PIP_2_‐PLC‐PKC pathway requires the mobilization of Ca^2+^ from stores. When included in the intracellular solution, the rapid calcium buffer BAPTA (10 mmol/L) prevented activation of the PKC pathway with OAG (Fig. 3C). We also tested the effectiveness of OAG in potentiating the mGluR6‐current in the presence of xestospongin C, an inhibitor of IP_3_ receptor‐mediated intracellular calcium release. In the presence of xestospongin C (1 *μ*mol/L), we observed no increase in the current amplitude (Fig. 3D), indicating that potentiation of mGluR6‐current requires the mobilization of Ca^2+^ from stores, a well described feature of the PIP_2_‐PLC‐PKC pathway. Taken as a whole, these data indicate that mGluR1 increased mGluR6‐evoked currents through the PKC pathway (Fig. 3E).

Furthermore, inhibition of PLC with U73122 (10 *μ*mol/L) prevented potentiation of mGluR6‐currents by mGluR1. When applied through the recording pipette, U73122 reduced the light‐evoked current by 41% (mean response amplitude in the presence of U73122 = 14 ± 1 pA; Fig. 3F, *n* = 4; mean current amplitude under control conditions = 24 ± 4 pA, *n* = 7, data not shown). This finding further supports the idea of ongoing activity of the PIP_2_‐PLC‐PKC pathway in the dark. Subsequent bath application of AIDA following dialysis of U73122 had no further effect on the current amplitude (Fig. 3F and G). Collectively, these results indicate that signaling modulation by mGluR1 requires activation of the PIP_2_‐PLC‐PKC pathway.

### mGluR1α modulates the rod‐RBC synapse postsynaptically in RBC dendrites

Our physiological results indicate that mGluR1 modulates RBC light responses through the PLC‐PKC pathway in the RBC dendrites. However, the work of Cai and Pourcho (1999) suggested that mGluR1 may be expressed in rod spherules where it would modulate this synapse presynaptically in the cat retina. To confirm that mGluR1 is present within RBC dendrites, we performed immunostaining using an antibody against mGluR1.

**Figure 4:**
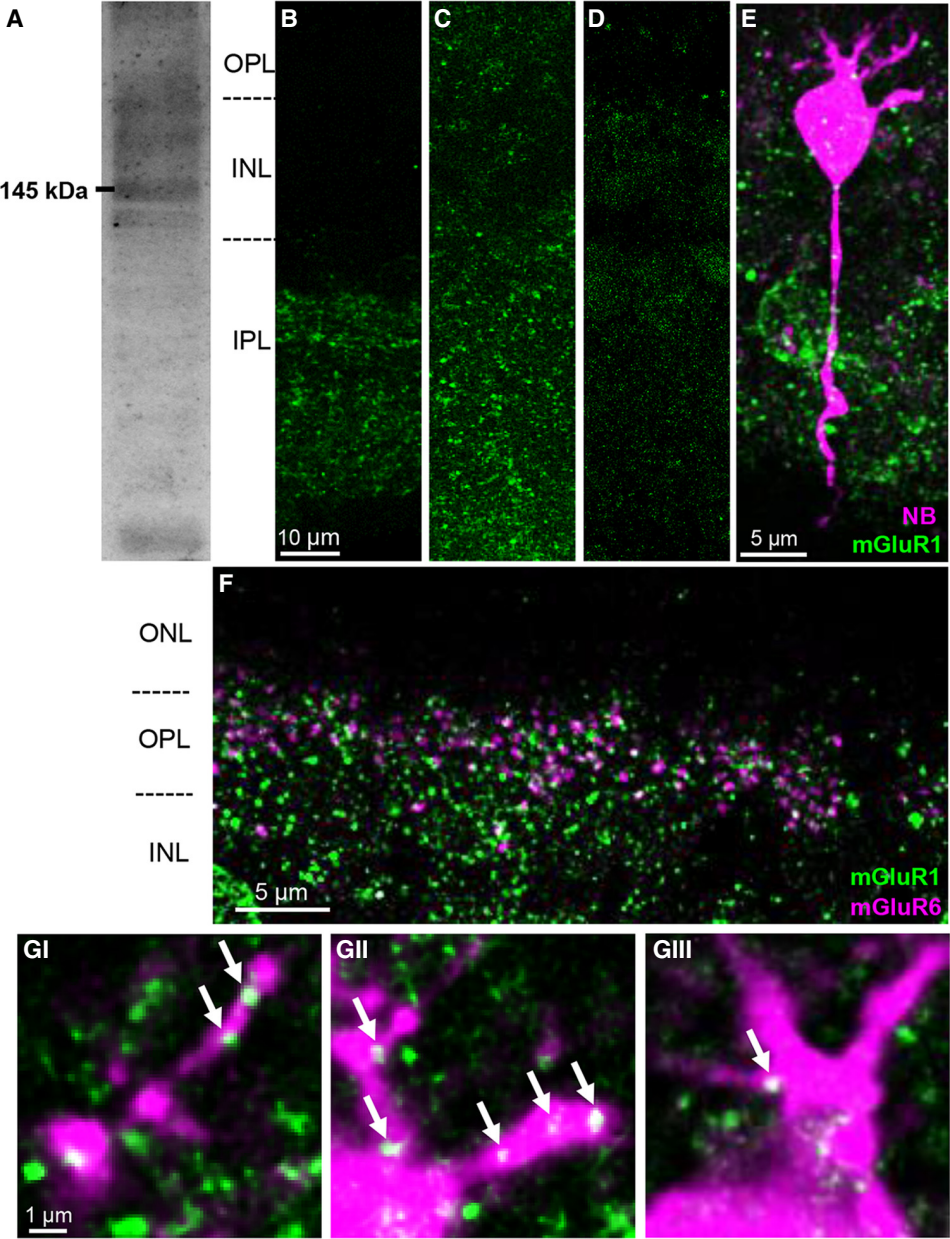
mGluR1 is expressed in the rod bipolar cell (RBC) dendrites. (A) A Western blot is shown indicating the mGluR1 band at ~145 kDa. (B) The mGluR1 antibody did not stain well when the tissue was fixed with 4% paraformaldehyde. (C) It stained retinal tissues when fixed with 2% paraformaldehyde after conducting antigen retrieval. (D) Control staining without the primary antibody. (E) A representative Neurobiotin filled RBC stained for mGluR1. (F) A wider view of mGluR1 staining revealing that mGluR1 puncta do not extend into the outer nuclear layer. (Gi‐Giii) mGluR1 puncta colocalized with RBC dendrites. Individual digital sections (0.3 *μ*m thick) are shown for a representative labeled cell (E). NB = Neurobiotin.

The mGluR1 antibody was authenticated via Western blot where the expected band is indicated at 145 kDa (Fig. 4A). While the predicted size of the protein is around 133 kDa, it is typically observed around 145 kDa possibly due to the heavy N‐linked glycosylation (Masu et al. 1991; Ayala et al. 2012). An additional lower band likely corresponds to the 100 kDa calpain‐truncated product observed by (Xu et al. 2007). Furthermore, Ayala et al. (2012) suggested that this mGluR1 antibody would not recognize its antigen under typical conditions of 4% paraformaldehyde fixation, and indeed we observed poor staining in this condition (Fig. 4B). To resolve this, we performed 2% paraformaldehyde fixation followed by antigen retrieval, which enhanced punctuate staining (Fig. 4C). We further verified the immunostaining specificity by omitting the primary antibody (Fig. 4D), which abolished labeling seen in Figure 4C.

We then examined whether mGluR1 is expressed in RBC dendrites. To do this, RBCs were injected with Neurobiotin and retinal tissues were fixed and stained with the mGluR1 antibody. While the staining appeared heavy at first, the distinct lack of labeling in the outer nuclear layer (ONL) (Fig. 4F) confirmed that this staining was not random noise. A representative Neurobiotin injected RBC is shown in Figure 4E; we found that mGluR1 puncta colocalized with RBC dendrites as is shown in single digital sections (0.3 *μ*m, Fig. 4Gi–Giii, *n* = 3 mice) of the representative cell shown in Figure 4E. Neurobiotin staining did not always reveal the dendritic compartment of RBCs, and these unfilled cells were excluded from data analysis. Nonetheless, mGluR1 puncta were observed in dendrites of all filled RBCs we examined (n=8 RBCs). Thus, consistent with the results of Koulen et al. (1997), our immunohistochemical data show that mGluR1 is present postsynaptically in RBC dendrites.

### mGluR1α activation is dependent on adaptation state

**Figure 5:**
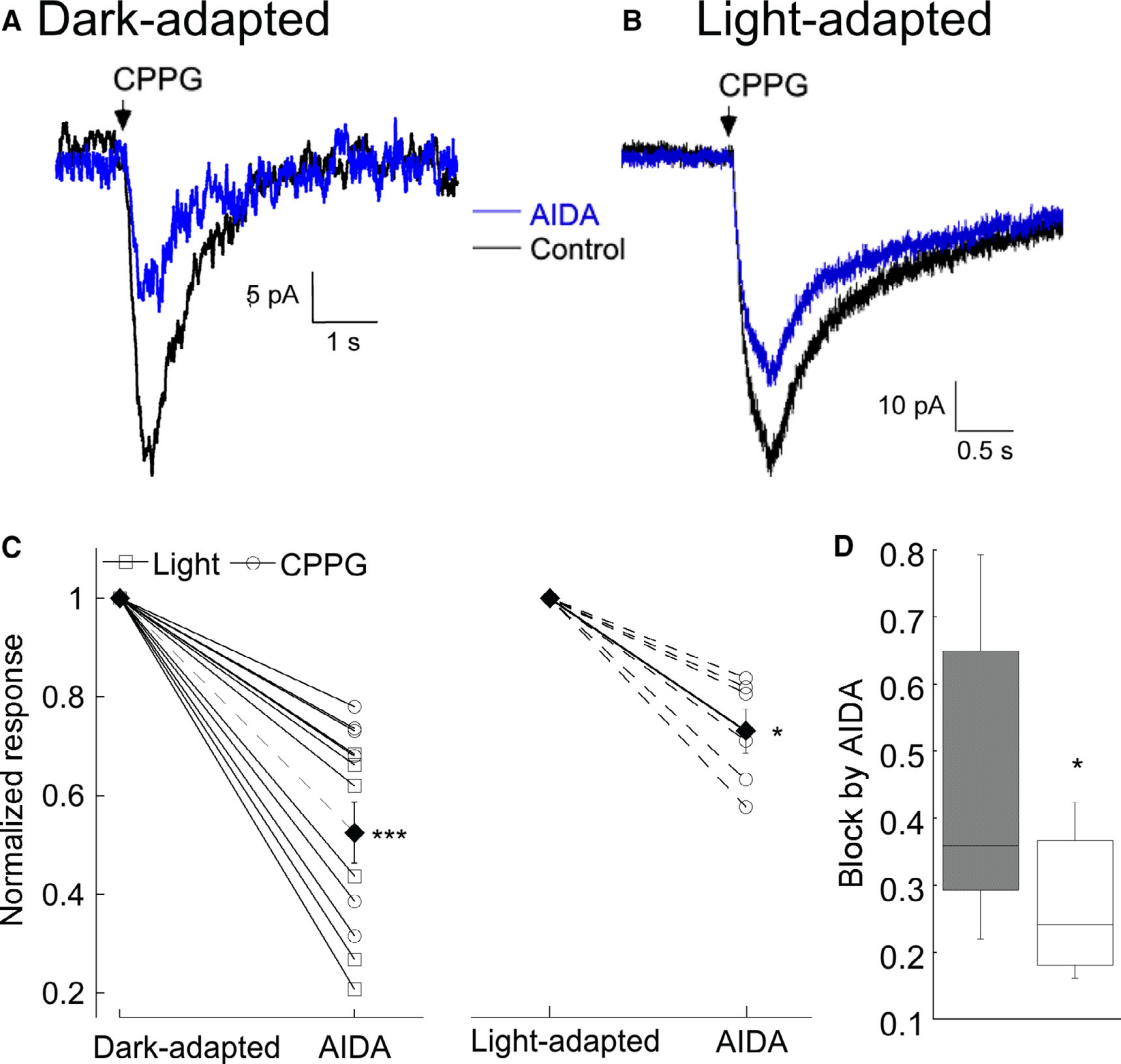
mGluR1 potentiation of mGluR6 current depends on adaptation state. (A) Example of the effect of the mGluR1 antagonist (RS)‐1‐Aminoindan‐1,5‐dicarboxylic acid (AIDA) on the (RS)‐α‐Cyclopropyl‐4‐phosphonophenylglycine (CPPG)‐evoked mGluR6‐current in the dark‐adapted state. (B) A similar experiment, except the recording was performed in a light‐adapted retina. (C) Summary plot of the mean ± SEM inhibition by AIDA (filled symbol) and in individual cells (open symbols) in dark‐adapted (left, *n* = 12) and light‐adapted (right, *n* = 7) conditions. Some experiments were carried out using light rather than CPPG to activate mGluR6 current in the dark‐adapted state. No significant difference was noted between these two groups, so they have been pooled (***indicates *P* < 0.0001 (dark‐adapted) and *indicates *P* = 0.014 (light‐adapted) before and after application of AIDA (single variable *t* test vs. 1). (D) Comparison of the reduction of mGluR6 current by AIDA in light‐ and dark‐adapted tissue (* indicates *P* = 0.014).

In dark‐adapted conditions, photoreceptors continuously release glutamate which activates mGluR1 to enhance mGluR6‐evoked currents. In contrast, signal enhancement by mGluR1 should be reduced in the light‐adapted conditions where ambient glutamate is low. To examine whether different light conditions change gain control via mGluR1, we compared responses in the dark and light‐adapted conditions. In the light‐adapted conditions, the light responses are absent, and so mGluR6 currents were evoked by the mGluR6 antagonists LY341495 (500 *μ*mol/L) or CPPG (600 *μ*mol/L) (Fig. 5). The RBC response to either antagonists is equivalent (*n* = 10 cells, data not shown). The initial response to CPPG in both adaptation states was nearly equivalent (dark‐adapted: 27 ± 4.6 pA, light‐adapted: 24 ± 6.5 pA). The application of the mGluR1 antagonist AIDA (100 *μ*mol/L) reduced the current to nearly 50% of CPPG‐evoked current in the dark‐adapted retina (Fig. 5A), similar to its effects on light‐evoked currents presented in Figure 1. In contrast, only AIDA slightly reduced the current in the light‐adapted tissues, although the decrease was statistically significant (Fig. 5B–D). The comparisons of the effect of blocking mGluR1 in the dark and light‐adapted states are summarized in Figure 5C and D. These results suggest that synaptic gain control by mGluR1 changes in scotopic and mesopic light conditions.

**Figure 6:**
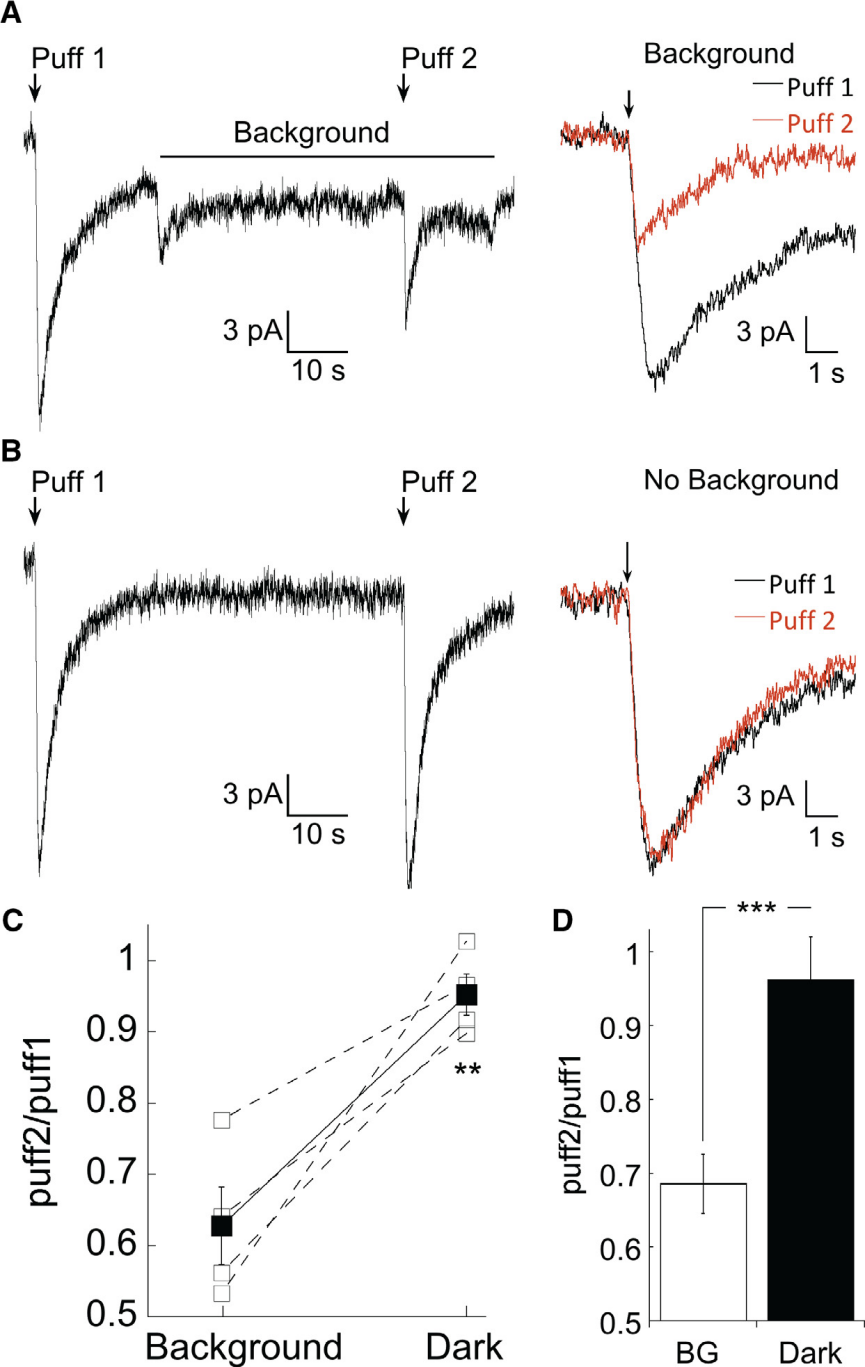
Dim backgrounds are sufficient to reduce mGluR1 potentiation. (A) Left: Application of an mGluR6 antagonist in the dark (puff 1) evokes mGluR6 current. A background light producing 72 R*/rod/s was then turned on and the mGluR6 antagonist was applied a second time (puff 2). L‐AP4 was absent from the bath, as mGluR6 receptors were activated by endogenous glutamate. Right: Puffs 1 and 2 from the same recording shown on an expanded time scale. The shift in baseline due to background illumination has been offset for clarity. (B) As in (A), but in the absence of a background. Puffs 1 and 2 evoke currents of identical amplitude. (A) and (B) are from the same cell. (C) Comparison of the amplitude of puffs 1 and 2 in the absence and presence of a background (*n *= 4, **indicates *P* = 0.002, paired *t* test). (D) Comparison of all trials in which the puff ratio was examined in the dark and in the presence of a steady background independent of specific cells (background, *n* = 12; dark, *n* = 9; *P* < 0.001).

Thus far we found that in the light‐adapted state, the absence of endogenous glutamate release from presynaptic photoreceptors results in a loss of mGluR1 activation and a reduction in the responsiveness of the mGluR6 transduction pathway. However, it is unclear if strong adaptation is required to turn off this pathway, or if more subtle background illumination could produce the same effect. To address this, we measured the size of the simulated light response in dark‐adapted retinas in the presence and absence of a dim background. We chose to use a pharmacological approach to activate the mGluR6 cascade rather than measuring the flash response because this approach allows us to selectively measure postsynaptic changes in gain without confounding variables such as rod adaptation. An example of this experiment is shown in Figure 6A. In darkness, we evoked a transient receptor potential cation channel subfamily M member 1 (Trpm1) current with the mGluR6 antagonist LY341495. A background was then applied to the retina, and the response to LY341495 was measured again in the presence of the background. A 30s background light that bleached less than 100 R*/rod/s reduced the amplitude of the simulated light response by approximately 50% (Fig. 6A). As we predicted, the peak of the Trpm1 current evoked by a puff of mGluR6 antagonist is reduced in the presence of a background, implying a reduction in the efficiency of Trpm1 channel opening following unbinding of glutamate from the mGluR6 receptor. We confirmed that this reduction is not due to the desensitization of Trpm1 but by the interleaving trials in which we elicited two simulated light responses 30 sec apart without applying a background light; under these conditions, there was no change in the current amplitude (Fig. 6B). These data are summarized in Figure 6C, which compares the puff response in the presence of the background normalized to the response in the dark for each cell, and in Figure 6D, in which the total number of trials across cells are averaged. The difference in the effectiveness of channel opening by an mGluR6 antagonist in darkness and the presence of a steady background was highly significant. These data are consistent with the idea that less mGluR1 activation during a steady background reduces postsynaptic gain by decreasing the overall efficiency of the mGluR6 cascade.

### PKCα expression changes in the light and dark‐adapted retina

In the retina, PKC expression has been shown to be dependent on the light level, although the effects of light are still unclear. Using an antibody against the hinge region of PKC*α*, Gabriel et al. (2001) showed that in the rat retina PKC*α* expression varied with the ambient light level, while no light dependency occurred when a PKC*α* antibody against the regulatory subunit was used. Xiong et al. (2015) also used the same hinge region antibody in the mouse retina to show that PKC*α* expression changes dependent on the light levels. However, these results were not consistent, and thus, we sought to clarify the state of PKC expression in varying light conditions in our mouse preparations.

**Figure 7:**
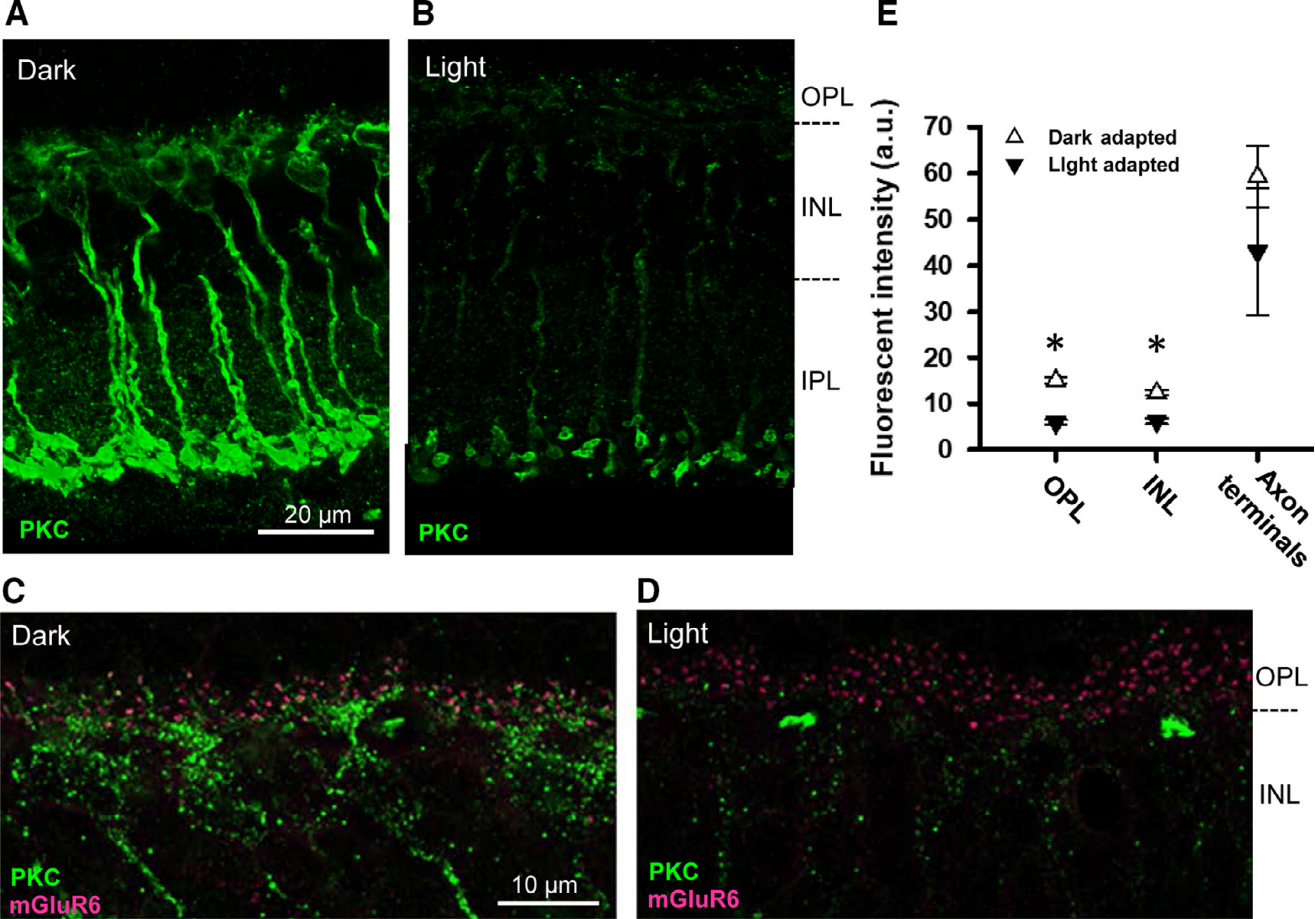
Protein kinase C*α* (PKC *α*) expression changes in light and dark‐adapted retina. (A and B) Immunostaining of cryosectioned mouse retinas from dark and light‐adapted animals (*N* = 3 each) for PKC *α*. (C and D) A 2× digitally zoomed image shows the intensity of PKC *α* in the outer nuclear layer and outer plexiform layer (OPL). mGluR6 is stained in magenta to indicate the location of dendritic tips. (E) PKC *α* fluorescent intensity varies between light and dark‐adapted conditions. PKC *α* expression was found to be more strongly expressed in the inner nuclear layer (INL) and OPL of the rod bipolar cells ( RBCs) in dark‐adapted tissue versus light‐adapted tissue (paired *t*‐tests, *P* < 0.001 for OPL,* P* < 0.05 for INL).

We observed that PKC expression was more robust from the RBC dendrites to the axon terminals in the dark‐adapted mice than in the light‐adapted animals (Fig. 7A–B). We also labeled the dendritic tips by costaining for mGluR6 and PKC in the light and dark conditions, but did not observe elevated PKC in the light‐adapted RBC dendritic tips (Fig. 7C and D) which has been previously suggested (Xiong et al. 2015). Quantification of the pixel intensity from the OPL, INL, and axon terminal regions confirmed that RBCs express higher levels of PKC in the OPL and ONL of dark‐adapted tissues (Fig. 7E, *N* = 3 for each light condition, *P* < 0.001 for OPL, *P* < 0.001 for INL, un‐paired *t* test). Taken together, these results are consistent with the idea that in the dark‐adapted state, high levels of PKC*α* are present in the dendrites of RBCs where they can mediate the positive effects of mGluR1 on gain control. Conversely, PKC*α* levels are low in the light‐adapted retina, consistent with our observation that mGluR1 has a reduced effect on gain control in this adaptation state.

### mGluR1 changes the direction of synaptic gain control in the light‐adapted conditions

**Figure 8:**
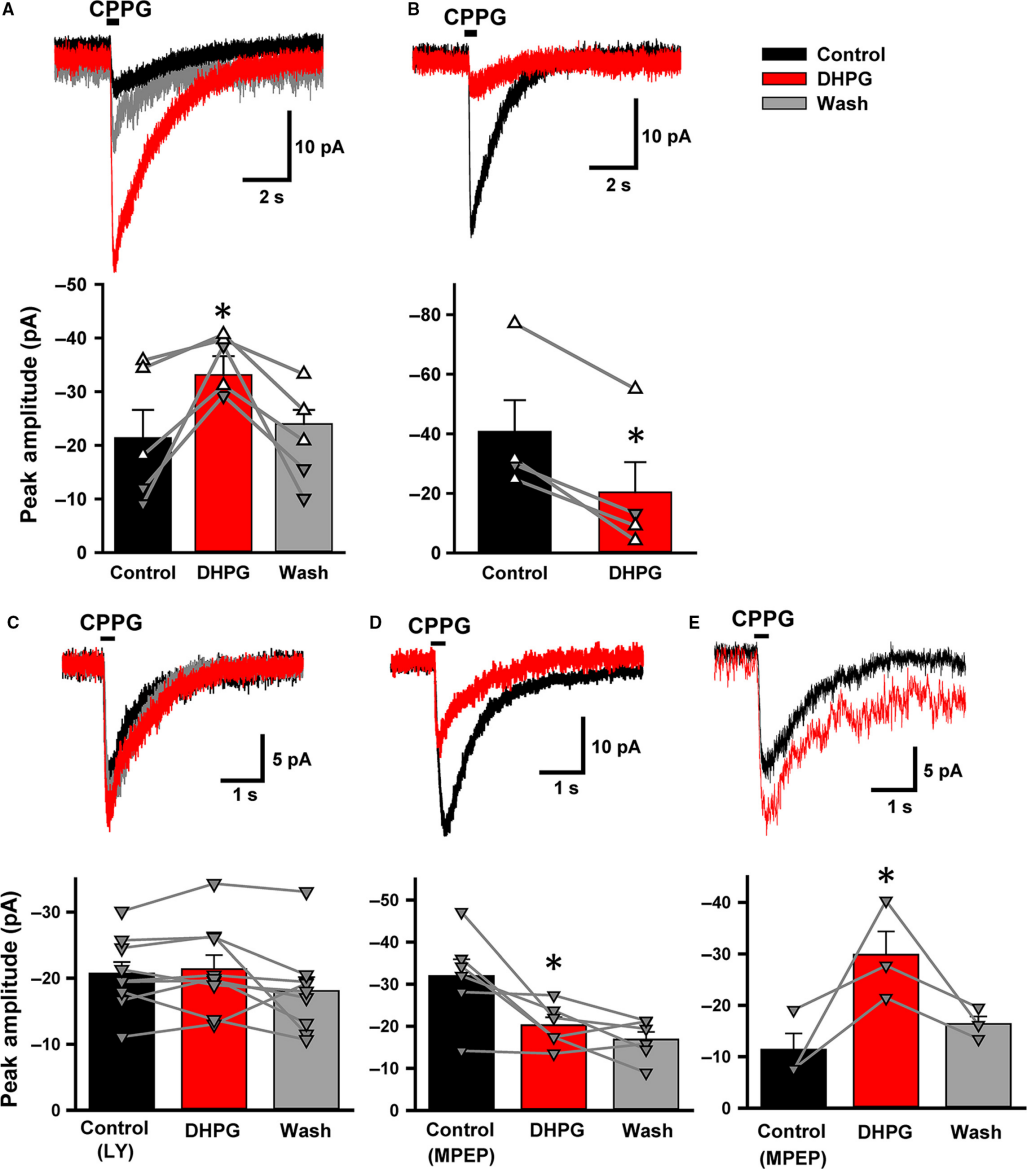
A group I mGluR agonist, 3,5‐Dihydroxyphenylglycine (DHPG), modulated mGluR6‐evoked currents in two ways. (A) Top: (RS)‐α‐Cyclopropyl‐4‐phosphonophenylglycine puff evoked an inward current in the rod bipolar cells ( RBCs) (black). The current was increased in the presence of DHPG (100 *μ*mol/L, red). Bottom: The summary graph shows currents increased by DHPG (*n* = 5, paired *t*‐test, *P* < 0.05). In this and following panels, open triangles (∆) indicate perforated patch and closed triangles (▼) indicate whole cell recordings (B) Top: DHPG sharply reduced the CPPG‐evoked currents. Bottom: The summary graph shows the current decreased by DHPG (*n* = 4, paired *t*‐test, *P* < 0.01). (C) Top: In the presence of LY367385 (100 *μ*mol/L) (black), DHPG (100 *μ*mol/L) did not change the mGluR6‐evoked current (red). Bottom: A summary graph showing the effects of LY367385 and DHPG together on evoked currents (*n* = 9, paired *t*‐test, *P* > 0.1). (D) Top: Pre‐application of 2‐Methyl‐6‐(phenylethynyl)pyridine hydrochloride (50 *μ*mol/L) did not prevent dual effects of DHPG. A decreased response and a summary graph are shown (top and bottom, respectively) (*n* = 6, paired *t*‐test, *P* < 0.05). (E) An increased response and a summary graph are shown (top and bottom, respectively) (*n* = 3, paired *t* test, *P* < 0.05)).

To directly address the possibility that decreased PKC expression at the dendrites of RBCs in the light‐adapted conditions might affect mGluR1 signaling, we activated mGluR1 with the agonist DHPG (100 *μ*mol/L). As with recordings made in dark conditions, intrapipette Ca^2+^ was buffered to near 0 mmol/L. We evoked mGluR6 currents with CPPG and applied DHPG to examine the effect of group I mGluRs. Surprisingly, DHPG enhanced the CPPG‐evoked currents in some RBCs (Fig. 8A), but decreased currents in others (Fig. 8B). Both effects were found to be significant (*n* = 5, *P* < 0.05 for increment RBCs; *n* = 4, *P* < 0.01 for decrement RBCs, paired *t* tests) and the inhibitory effects of DHPG were not due to rundown of RBC responses as the decrement of EPSC amplitude did not begin until application of DHPG (data not shown). Additionally, the dual effects of DHPG also occurred in the perforated patch configuration (open triangles, Fig. 8A and B, bottom panels). Morphological differences were not observed between these two groups of RBCs.

DHPG activates both group I mGluR1 and mGluR5, raising the possibility that mGluR5 may play a role in decreasing CPPG‐evoked currents. However, both the incremental and decremental changes in response amplitude produced by DHPG were blocked by the mGluR1 antagonist, LY367385 (Fig. 8C). Conversely, in the presence of the mGluR5 antagonist MPEP, DHPG similarly showed both the incremental and decreasing effects on the mGluR6‐current (Fig. 8D and E), suggesting that mGluR1 alone induced both the increment and the decrement of mGluR6 currents.

**Figure 9:**
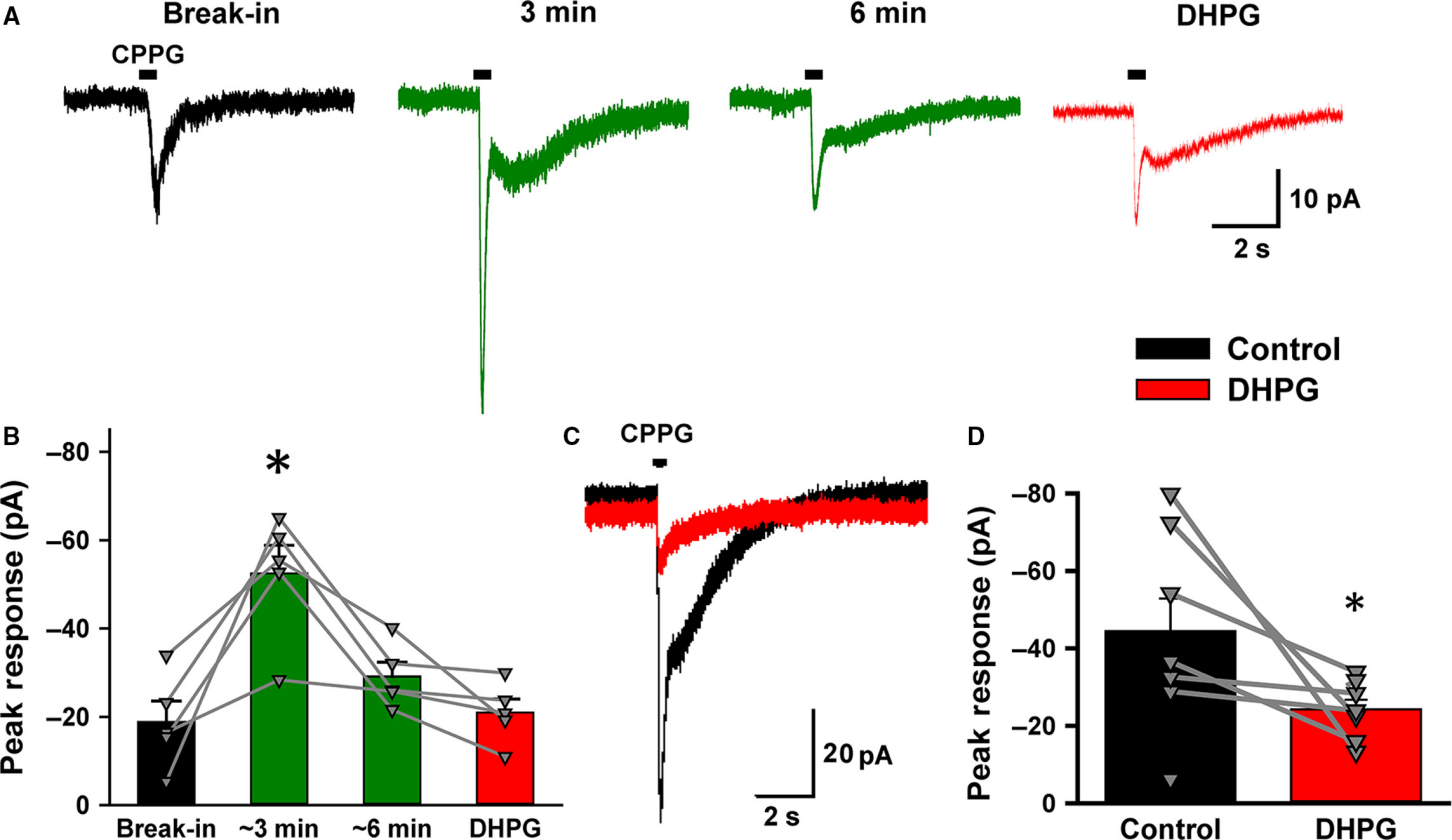
A protein kinase C (PKC) antagonist and high intracellular Ca^2+^ concentration mediated the dual effects of mGluR1 in the light. (A) A PKC antagonist, Go6976 (1 *μ*mol/L) was included in the pipette solution. (RS)‐α‐Cyclopropyl‐4‐phosphonophenylglycine (CPPG)‐evoked, mGluR6‐current at break‐in (black), 3 min (dark green), and 6 min (dark green) after whole cell configuration started. Subsequent application of 3,5‐Dihydroxyphenylglycine (DHPG) did not change the mGluR6‐current (red). (B) The summary graph shows the time course of PKC effects on CPPG‐evoked currents. Individual cells are plotted in grey. For break‐in versus 3 min: *n* = 5, paired *t* test, *P* < 0.05. For 6 min versus DHPG:* n* = 5, *P* > 0.05. (C) A representative trace with 1 mmol/L Ca^2+^ included in pipette solution (black) which is reduced by 100 *μ*mol/L DHPG (red). (D) A summary graph showing the effects of DHPG on CPPG‐evoked currents in the presence of 1 mmol/L Ca^2+^. The majority of the current decreased by DHPG (solid grey lines), while one cell moderately increased (dashed grey lines). For the decreased currents: *n* = 6, paired *t* test, *P* < 0.05.

To test if both actions of DHPG involved in the PLC‐PKC signaling pathway, we included the PKC inhibitor Go6976 (1 *μ*mol/L) in the recording pipette. Immediately after break‐in, the amplitude of CPPG‐evoked mGluR6‐current was −19 ± 4 pA (*n* = 5). After several minutes of whole cell recording, the amplitude transiently increased to −53 ± 6 pA (*P* < 0.05, *n* = 5, paired *t* test), and then gradually decreased back to the control level (Fig. 9A–B, *P* < 0.05, break‐in vs. ~3 min: *P* = 0.07, break‐in vs. ~6 min, *n* = 5, paired *t* test). Subsequent application of DHPG did not change the CPPG‐current (*P* > 0.05, ~6 min vs. DHPG, *n* = 5, paired *t* test), indicating that both the incremental and decrement effects were evoked by mGluR1 and were mediated through the PKC pathway.

We wondered what induced the distinct modulation of mGluR6 current by mGluR1 in the light‐adapted conditions. We increased intracellular free Ca^2+^ to 1 mmol/L, and examined the effect of DHPG. While with a 0 mmol/L Ca^2+^ pipette DHPG either increased or decreased 50% of RBCs (*n* = 5 each, Fig. 8A–B), with 1 mmol/L Ca^2+^ in the pipette, DHPG decreased the current in 7 of 8 RBCs (Fig. 9C–D). This change in DHPG effect was found to be significant (unpaired *t* test, *P* < 0.05). Thus, elevated free Ca^2+^ in RBCs biases the mGluR1 activation toward a decrease in synaptic gain between rods and RBCs.

## Discussion

We found that mGluR1 regulates the synaptic gain of rod–RBCs through the PLC‐PKC signaling pathway. In dark conditions, mGluR1 increased the gain of synaptic transmission through activation of the PLC‐PKC pathway. In the light, mGluR1 exhibited diverse effects that were influenced by levels of intracellular PKC and Ca^2+^.

### The mGluR6‐TrpM1 complex and group I mGluRs in RBC dendrites

Both mGluR1 and mGluR5 are group I mGluRs that couple to phosphoinositide hydrolysis (Masu et al. 1991; Abe et al. 1992; Aramori and Nakanishi 1992) and activate the PLC‐IP_3_‐PKC pathway, increasing membrane excitability. Previous studies place mGluR1 and mGluR5 at RBC dendrites in the rat retina (Koulen et al. 1997, 2005), although a presynaptic locus for mGluR1 in cat retina has also been proposed (Cai and Pourcho 1999). In addition, mGluR6, a group III mGluR, makes a complex with TrpM1 and other molecules to serve as the primary glutamate receptor that conveys signaling between rods and RBCs (Morgans et al. 2007, 2009; Cao et al. 2009; Koike et al. 2010; Shen et al. 2009; Ray et al. 2014; Tummala et al. 2014; Ramakrishnan et al. 2015). Although Rampino and Nawy (2011) found that TrpM1‐evoked currents were regulated by the PIP_2_‐PLC‐PKC signaling pathway, gain control mechanisms by group I mGluRs have not been reported.

A key feature of the mGluR6 pathway is that binding of glutamate to mGluR6 results in closure of the TrpM1 channel. If glutamate binds to both mGluR1 and mGluR6, then mGluR1 activation would be expected to potentiate a current that is simultaneously suppressed by mGluR6. This would appear to be an ineffective design. The resolution to this paradox may lie in the different temporal properties of the mGluR1 and mGluR6 pathway. The mGluR6 pathway necessarily operates on the order of milliseconds, matching the kinetics of the rod light response (Berntson et al. 2004). It is thought that signaling of the mGluR6 pathway relies on the direct interaction of G*βγ* and G*α*_o_ subunits with the TrpM1 channel (Martemyanov and Sampath 2017); in contrast, group I mGluRs require second‐messenger systems to couple to their downstream targets. Additionally, the kinetics of mGluR1 activation/inactivation may be slower, integrating over longer times, rather than responding to rapid changes in transmitter release. Finally, our immunohistochemistry suggests that mGluR1 may not be expressed at the dendritic tips, but rather at a small distance from the tips (Fig. 4); this is consistent with the idea of mGluR1 as an ambient glutamate sensor in contrast to mGluR6 acting as an instantaneous glutamate sensor. Regardless, the experiments described here suggest that the mGluR1 pathway can be downregulated by an increase in background illumination within 1 min (Fig. 6), but we did not attempt to further resolve the kinetics of this pathway.

All together, our results in mouse RBCs show that mGluR1 increases the synaptic gain in the dark; however, mGluR5 did not play a role in modulating mGluR6‐current in RBCs (Figs. 2 and 8). While mGluR1 was found in rod spherules in the cat retina (Cai and Pourcho 1999), postsynaptic expression has been suggested in the mouse retina (Koulen et al. 2005). Our immunohistochemical data (Fig. 4) and our physiological observations (Fig. 3) strongly support a postsynaptic locus of action. Moreover, if mGluR1 or mGluR5 was present presynaptically in rods, their activation would depolarize the rod terminal, increasing the amount of glutamate released into the synapse. It is also possible that postsynaptic group I mGluRs could alter membrane potential of the RBC. However, we did not observe any shift in membrane potential with LY367385 or MPEP application (see Results section), and so we ruled out the possibility of a presynaptic mechanism of action for mGluR1 and mGluR5.

### mGluR1 increased synaptic gain in the dark‐adapted conditions

In the dark‐adapted conditions, mGluR1 antagonists solely reduced the light‐evoked responses (Fig. 1), indicating that mGluR1 increased the gain of mGluR6‐mediated synaptic responses. Human psychophysical studies indicate that the rod pathway is able to detect extremely dim light, requiring only approximately five rods to absorb a single photon for a detectable response (Hecht et al. 1942), and amplification in the rod pathway is a key component for reliably transmitting these signals. Interpreting the benefit of activating the mGluR1 pathway is therefore fairly straightforward: maintaining activation of the mGluR1 pathway in the dark keeps the synapse primed to transmit small signals that could otherwise be lost as noise. The continuous activation of mGluR1 might induce desensitization (Kawabata et al. 1996; Uchino et al. 2004). However, our results did not reveal any desensitization because mGluR1 antagonists always decreased the mGluR6‐evoked responses (Fig. 1), indicating that mGluR1 provides a reliable gain mechanism in dark conditions.

Consistent with our physiological results, expression of PKC*α* in dendrites and somas of RBCs was higher in the dark than in the light conditions. Although we used a PKC*α* antibody against the hinge region of PKC*α* that was used by Gabriel et al. (2001) and by Xiong et al. (2015), our results were not exactly consistent with their results. Gabriel et al. (2001) used the rat retina and showed that PKC*α* expression increased in the light conditions and decreased in dark conditions. The different results might be attributable to species differences. The results of Xiong et al. (2015), which used the mouse retina, were very similar to ours except that they observed RBC dendritic tips highlighted in the light conditions. We carefully examined RBC dendritic tips by co‐labeling with mGluR6 (Fig. 7) and with CtBP (data not shown); however, we never found that the tips were highlighted in any light conditions. One possibility for the difference observed in our results versus those of Xiong et al. (2015) could be the length of dark adaptation of animals prior to immunostaining.; Gabriel et al. (2001) demonstrated that beyond 12 h of dark adaption, PKC expression increases regardless of light stimulation; as such, we dark‐adapted the mice for 12 h while Xiong et al. (2015) used 24 h. Regardless, except for the dendritic tips, our overall results are consistent with those of Xiong et al. (2015) where RBCs exhibited greater PKC*α* expression in the dendrites and soma of RBCs in dark compared to light conditions.

### mGluR1 exhibited mixed synaptic gain changes in the mesopic conditions

In the mesopic light conditions, mGluR1 still modulated mGluR6‐activated currents, but the effect was different from that observed in the dark‐adapted conditions. Surprisingly, mGluR1 increased mGluR6‐currents in some RBCs, but in other RBCs, mGluR1 decreased mGluR6‐mediated current (Fig. 8). The decrement of the current is found in mesopic conditions where the rod‐signaling approaches the saturation and the cone signaling becomes functional. During this phase from rod to cone signaling dominancy, shutting off the transmission between the rod and RBC by mGluR1 should contribute to a smooth transition. Upon switching to higher background light levels, the output of RBCs is suppressed via both extrinsic and intrinsic mechanisms, such as depletion of transmitter from the RBC terminals (Dunn and Rieke 2008; Jarsky et al. 2011; Oesch and Diamond 2011), rod–cone crossover (Marc et al. 2014; Lauritzen et al. 2016), and inhibition by glutamate transporters at RBC terminals (Ichinose and Lukasiewicz 2012). Here we identify an additional intrinsic mechanism, whereby mGluR1 serves decreases in gain between rods and RBC during the transition to cone dominated signaling. As elevated intracellular Ca^2+^ biased the mGluR1 pathway toward the reducing synaptic gain (Fig. 9 and below), it may be that Ca^2+^ influx from TrpM1 channels contribute to this switch from high to low gain.

In the mesopic conditions, mGluR1 alone controlled the synaptic gain in two ways (Figs. 2 and 8). Interestingly, both the effects were mediated through the PKC signaling pathway because DHPG did not modulate the mGluR6‐current after diffusion of Go6976 in the intracellular space (Fig. 9). Go6976 is a specific inhibitor of PKC; however, it is not specific to PKC*α*, but also to PKC‐*β*,* ε*,* δ*, and *ζ* (Martiny‐Baron et al. 1993). Potentially, the dual effects of mGluR1 are mediated through different isozymes of PKC. Indeed, studies of other mammalian species such as the cat retina have found multiple isozymes of PKC to be expressed in RBC besides PKC*α* (Fyk‐Kolodziej et al. 2002). However, pursuing various PKC signaling pathways is beyond the scope of this study. We moreover found that high Ca^2+^ intracellular solution biased mGluR1 modulation toward a decrement of mGluR6‐currents (Fig. 9), suggesting that it is most likely the Ca^2+^‐sensitive PKC isozymes that mediate mGluR1 modulation.

During the transition to mesopic light, it is likely that mGluR1 is still active. It has been proposed that synaptic glutamate concentrations can be as high as 100 *μ*mol/L at the rod‐RBC synapse (Hasegawa et al. 2006) in the dark, and that glutamate release is reduced by approximately 90% in the mesopic ambient light (Witkovsky et al. 1997). One would then infer that at mesopic light levels, there is still around 10 *μ*mol/L of glutamate present. mGluR1 is known to have an EC50 of 7.5 *μ*mol/L for glutamate, and should be active over a similar light range as mGluR6 (Marcaggi et al. 2009). Furthermore, Yin et al. (2006) suggested that rods are still functional even during daytime light levels. Similarly, mGluR6 has been shown to have an apparent EC50 for glutamate of 10 *μ*mol/L (de la Villa et al. 1995), and is known to be active in RBCs up to mesopic levels. Thus, due to its high affinity for glutamate, mGluR1 activity is still expected to be active moving into the mesopic light range. However, reduced levels of PKC*α* may lead to a switch in gain control.

**Figure 10:**
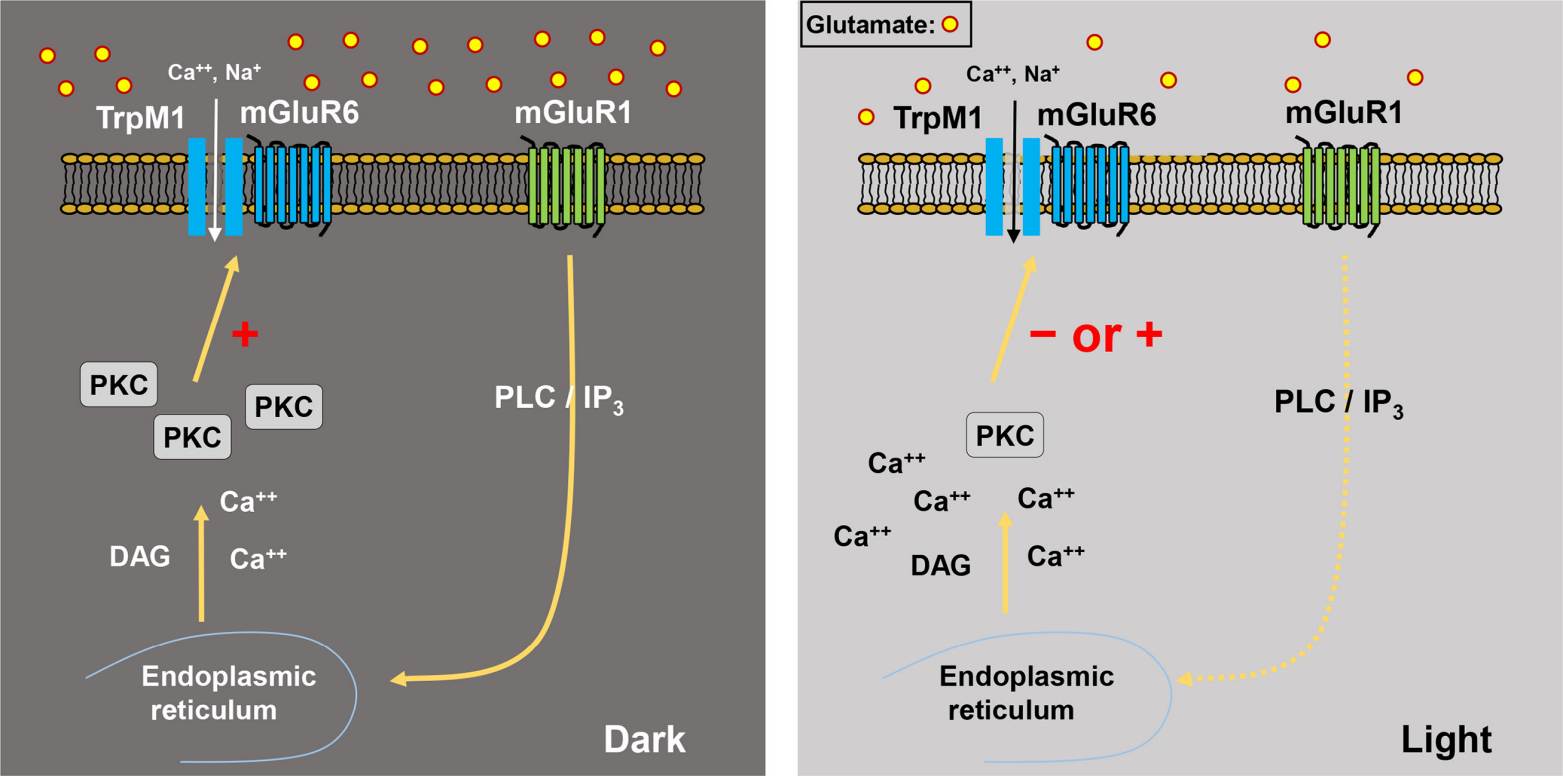
Schematic models of mGluR1 modulation of the mGluR6‐TrpM1 complex. (Dark) mGluR1‐mediated signaling activates the phospholipase C‐IP3‐protein kinase C pathway to amplify mGluR6‐evoked responses in the dark. (Light) Elevated intracellular Ca^2+^ blocks or reverses the effect of mGluR1 signaling on mGluR6‐mediated responses.

In conclusion, we found that mGluR1 serves as a gain control mechanism at the rod–rod bipolar cell synapse. In the dark, continuously released glutamate from rods activates mGluR1 in RBC dendrites, activating the PLC‐PKC*α* pathway to amplify light‐evoked responses (Fig. 10). In contrast, in the mesopic conditions, mGluR1 could activate different PKC pathways that suppress the synaptic responses.

## Conflict of Interest

The authors declare no competing financial interests.
